# Effects of Intercostal Nerve Cryoablation on Pain Control and Pulmonary Recovery after Open Aortic Repair via Left Thoracotomy

**DOI:** 10.5761/atcs.oa.25-00127

**Published:** 2025-10-23

**Authors:** Junji Nakazawa, Yutaka Iba, Tomohiro Nakajima, Tsuyoshi Shibata, Ayaka Arihara, Kenichi Kato, Kei Mukawa, Masato Yonemori, Shigeki Komatsu, Nobuyoshi Kawaharada

**Affiliations:** Department of Surgery, Division of Cardiovascular Surgery, Sapporo Medical University, Sapporo, Hokkaido, Japan

**Keywords:** aortic aneurysm surgery, intercostal nerve cryoablation, postoperative pain control, pulmonary recovery, opioid consumption

## Abstract

**Purpose:**

The aim of this study was to evaluate the impact of intercostal nerve cryoablation on postoperative pain, opioid usage, and lung expansion after open aortic repair via left thoracotomy.

**Methods:**

This retrospective study included 62 patients who underwent aortic repair via left thoracotomy between 2017 and 2023. Patients were divided into cryoablation (n = 32) and non-cryoablation (n = 30) groups. Pain was assessed using the Numerical Rating Scale (NRS), and lung volume was measured using computed tomography 1 week postoperatively.

**Results:**

The cryoablation group showed significantly lower mean NRS scores (1.7 vs. 2.4, *p* <0.01) and lower opioid consumption (6.2% vs. 56.6%, *p* <0.01). The left lung volume ratio was significantly higher in the cryoablation group (72.3% vs. 62.4%, *p* = 0.05).

**Conclusions:**

Intercostal nerve cryoablation effectively reduces postoperative pain and opioid consumption and enhances pulmonary expansion after left thoracotomy. This technique may offer a favorable analgesic option in thoracic aortic surgery.

## Introduction

Despite the widespread adoption of thoracic endovascular aortic repair, open surgical repair continues to play an essential role, particularly in patients with thoracoabdominal aneurysms or anatomies unsuitable for endovascular intervention.^1)^ However, this surgery is associated with significant postoperative pain due to its invasive nature, particularly when a left thoracotomy is required. Additionally, conventional analgesic regimens are often inadequate for managing the intense pain experienced after such procedures.^2–4)^

Excessive postoperative pain can suppress deep breathing and coughing, thereby increasing the risk of pulmonary complications such as atelectasis and pneumonia. Effective pain control is, therefore, not only essential for patient comfort but also critical for optimizing surgical outcomes.^5,6)^

In addition to conventional analgesics, common strategies for postoperative pain management include systemic opioid usage, epidural anesthesia, and intercostal nerve blocks.^7,8)^ However, each of these approaches has several limitations. To address these challenges, we have adopted intercostal nerve cryoablation since 2019 as a potentially safer and more effective method of achieving prolonged postoperative analgesia. This technique, previously reported as being effective in thoracic procedures such as pectus excavatum repair and lung resection, offers sustained pain relief with minimal systemic side effects.^9,10)^

In this study, we evaluated the efficacy of intercostal nerve cryoablation in reducing postoperative pain, minimizing opioid usage, and promoting pulmonary recovery in patients undergoing open aortic repair via left thoracotomy.

## Materials and Methods

### Study design and patients

This retrospective observational study included 72 patients who underwent thoracic or thoracoabdominal aortic aneurysm (TAA or TAAA) repair via left thoracotomy at our institution between January 2017 and July 2023. After excluding cases involving emergency surgery or infected aneurysms, 62 patients were enrolled and assigned to either the cryoablation group or the non-cryoablation group. Group allocation was determined based on the time period during which surgery was performed. Intercostal nerve cryoablation was introduced at our institution in 2019 and subsequently incorporated into our routine perioperative protocol. Therefore, all patients who underwent open thoracic aortic surgery via left thoracotomy after 2019 received cryoablation.

### Surgical technique and cryoablation

**Fig. 1:**
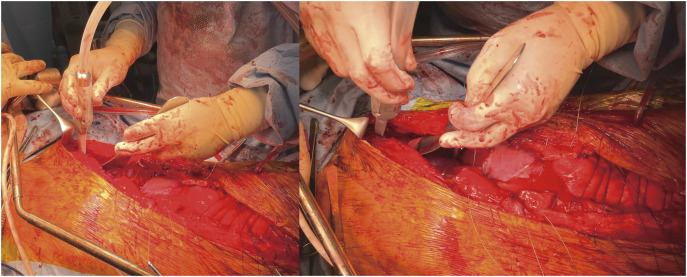
Intercostal nerve cryoablation was performed during aortic repair via left thoracotomy. At the end of the procedure, the cryoprobe tip, cooled to approximately −60°C, was applied to the intercostal nerves for about 2 min while protecting the lung with a spatula. Patient consent for publication of this intraoperative photograph was obtained.

Thoracotomy was performed through the 4th to 6th intercostal spaces using a 20–35 cm incision, depending on the location and size of the aneurysm. All procedures were conducted under single-lung ventilation of the right lung. After 2019, intercostal nerve cryoablation was performed using a reusable cryoprobe system (Freeze Stick M Type B; Shiraimatsu Corporation, Osaka, Japan), which utilizes the Joule–Thomson effect with compressed carbon dioxide gas to achieve localized freezing to approximately −60°C at the end of surgery (**Figs. 1A** and **1B**). The device was applied for approximately 2 min to the intercostal nerves corresponding to the thoracotomy site. To prevent tissue injury, warm water was applied to the probe prior to its removal.

### Postoperative pain management

Both before and after the introduction of cryoablation, all patients received acetaminophen and tramadol hydrochloride as scheduled, based on body weight. Nonsteroidal anti-inflammatory drugs were avoided as much as possible due to the risk of renal dysfunction. A fentanyl patch was applied as an opioid analgesic when pain was not adequately controlled with acetaminophen and tramadol hydrochloride. Epidural anesthesia was not used because of the potential risk of epidural hematoma.

### Outcome measures

**Fig. 2:**
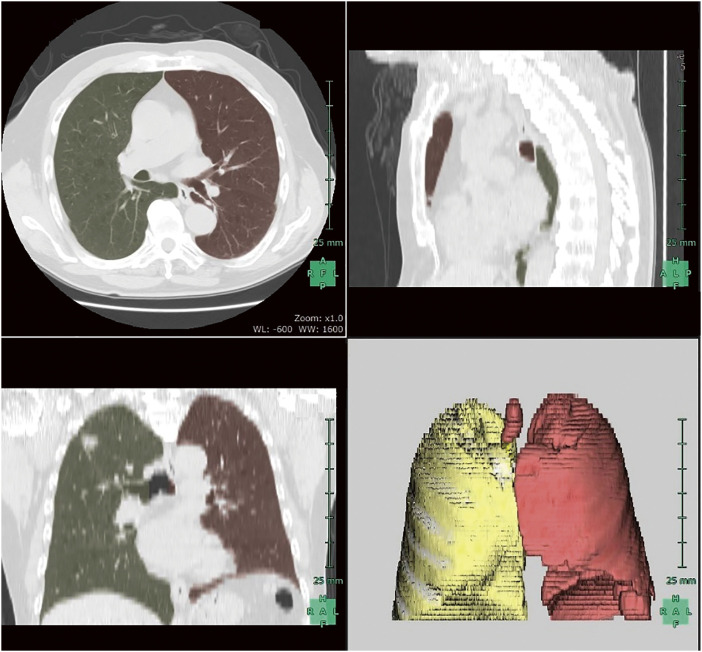
Lung volume assessment using SYNAPSE VINCENT (Fujifilm Co.). Lung fields were automatically identified based on computed tomography images, and 3-dimensional reconstructions were generated. Following manual verification, total and left lung volumes were calculated.

The primary outcome was postoperative pain, assessed using the mean Numerical Rating Scale (1–10) score over the first 5 postoperative days. Secondary outcomes included the frequency of opioid use, defined as the proportion of patients who required opioid analgesics during the postoperative period, and lung volume assessed by computed tomography (CT). Lung volume was calculated using the Synapse Vincent (Fujifilm Co., Tokyo, Japan) image analysis system, which automatically identifies lung fields and computes total volume (**Fig. 2**).

### Statistical analysis

Statistical analyses were performed using The Bell Curve for Excel (Social Survey Research Information Co., Ltd., Tokyo, Japan). Comparisons between groups were made using Student’s *t*-test for continuous variables and the chi-squared test for categorical variables. A *p*-value of <0.05 was considered statistically significant.

## Results

### Patient characteristics

A total of 62 patients were included in the study, with 32 patients in the cryoablation group and 30 in the non-cryoablation group. Baseline patient characteristics are shown in **Table 1**. There were no significant differences between the 2 groups in terms of age, sex, body mass index, body surface area, or comorbidities such as hypertension, dyslipidemia, diabetes mellitus, renal dysfunction, chronic obstructive pulmonary disease, and hemodialysis. The smoking rate was higher in the non-cryoablation group (73.3%) compared to the cryoablation group (40.6%) (*p* = 0.01). However, no significant differences were observed in EuroSCORE II, aneurysm type (TAAA vs. TAA), number of intercostal spaces resected, operation time, cardiopulmonary bypass time, or lowest intraoperative rectal temperature.

**Table 1:** Baseline patient characteristics

Variable	Cryoablation (n = 32)	Non-cryoablation (n = 30)	*p*-Value
Age (years)	64.3 ± 14.8	61.2 ± 12.2	0.36
Male, n (%)	26 (81.2)	22 (73.3)	0.54
Body mass index (kg/m^2^)	22.6 ± 3.9	23.2 ± 4.5	0.56
Body surface area (m^2^)	1.69 ± 0.16	1.74 ± 0.24	0.36
Hypertension (%)	22 (68.7)	22 (73.3)	0.78
Dyslipidemia (%)	9 (28.1)	6 (20.0)	0.55
Diabetes mellitus (%)	4 (12.5)	4 (13.3)	1
Renal dysfunction (%)	5 (15.6)	8 (26.7)	0.36
COPD (%)	15 (46.8)	11 (36.6)	0.45
Hemodialysis (%)	0	1 (3.3)	0.48
Smoking (%)	13 (40.6)	22 (73.3)	0.01
EuroSCORE II	6.1 ± 2.3	6.0 ± 2.0	0.82
TAAA (%)	22 (68.7)	23 (76.6)	0.57
TAA (%)	10 (31.3)	7 (23.4)	0.57
Diaphragmatic incisions (%)	23 (71.9)	24 (80.0)	0.77
Number of resected intercostal spaces	1.9 ± 1.0	2.1 ± 1.5	0.76
Operation time (min)	404 ± 117	366 ± 123	0.97
Cardiopulmonary bypass time (min)	155 ± 54	130 ± 68	0.11
Lowest intraoperative rectal temperature (°C)	33.4 ± 2.6	33.0 ± 5.0	0.71

Demographic and perioperative characteristics of patients who underwent aortic repair via left thoracotomy, comparing those with and without intercostal nerve cryoablation. Values are presented as mean ± standard deviation or number (percentage), as appropriate. There were no significant differences between groups except for smoking status, which was higher in the non-cryoablation group.

TAA: tumor-associated antigen; COPD: chronic obstructive pulmonary disease; TAAA: thoracoabdominal aortic aneurysm

### Postoperative outcomes

Patients’ postoperative outcomes are summarized in **Table 2**. Regarding the primary outcome, the cryoablation group showed significantly lower pain scores compared to the non-cryoablation group, both on postoperative day 1 (1.7 ± 1.0 vs. 2.8 ± 1.2, *p* <0.01) and as the mean score over postoperative days 1–5 (1.7 ± 0.9 vs. 2.4 ± 0.8, *p* <0.01). The rate of opioid usage was also significantly lower in the cryoablation group (6.2%) compared to the non-cryoablation group (56.6%, *p* <0.01). However, no significant differences were observed between the groups in terms of intensive care unit stay, postoperative hospital stay, or mechanical ventilation time.

**Table 2:** Postoperative outcomes

Variable	Cryoablation (n = 32)	Non-cryoablation (n = 30)	*p*-Value
ICU stay (days)	1.8 ± 1.4	1.3 ± 1.0	0.17
Postoperative hospital stay (days)	24.8 ± 10.6	29.3 ± 17.2	0.22
Mechanical ventilation time (hours)	19.8 ± 27.5	13.3 ± 17.0	0.27
Reintubation (%)	2 (6.2)	0	0.49
Postoperative pneumonia (%)	0	0	1.0
Postoperative paraplegia (%)	1 (3.1)	2 (6.6)	0.60
Pain score (Numerical Rating Scale, 1–10)			
Postoperative day 1	1.7 ± 1.0	2.8 ± 1.2	<0.01
Mean score (postoperative days 1–5)	1.7 ± 0.9	2.4 ± 0.8	<0.01
Opioid use rate (%)	2 (6.2)	17 (56.6)	<0.01
Preoperative lung volume (mL)			
Bilateral lung volume	4032 ± 901	4565 ± 1106	0.04
Left lung volume	1601 ± 508	1948 ± 695	0.03
Lung volume at 1 week postoperatively (mL)			
Bilateral lung volume	3199 ± 904	3391 ± 1073	0.44
Left lung volume	1131 ± 415	1235 ± 577	0.42
Ratio of bilateral lung volumes at 1 week (%)	79.5 ± 17.2	73.6 ± 11.9	0.12
Ratio of left lung volume at 1 week (%)	72.3 ± 21.1	62.4 ± 17.2	0.05

Postoperative outcomes comparing the cryoablation and non-cryoablation groups. Pain scores were assessed using a Numerical Rating Scale (1–10), where 0 indicates no pain and 10 represents the worst imaginable pain. Lung volumes were measured using computed tomography, and volume ratios represent the proportion of postoperative to preoperative lung volumes. Data are shown as mean ± standard deviation or number (percentage), as appropriate.

ICU: intensive care unit

Regarding lung volume, although preoperative lung volumes were significantly larger in the non-cryoablation group, no significant differences were observed in postoperative lung volumes between the 2 groups at 1 week. The ratio of bilateral lung volumes at 1 week postoperatively to preoperative values was not significantly different between the groups (79.5% ± 17.2% vs. 73.6% ± 11.9%, *p* = 0.12). However, the ratio of left lung volumes at 1 week postoperatively was significantly higher in the cryoablation group (72.3% ± 21.1%) than in the non-cryoablation group (62.4% ± 17.2%, *p* = 0.05), suggesting improved pulmonary expansion on the operative side.

## Discussion

At our institution, intercostal nerve cryoablation was implemented to enhance postoperative pain management, as informed by previous reports.^2–4,7–10)^ This study confirmed that intercostal nerve cryoablation significantly reduces postoperative pain and opioid consumption in patients undergoing aortic repair via left thoracotomy. In addition to these analgesic effects, cryoablation also contributed to improved left lung expansion, as assessed by CT at 1 week postoperatively, suggesting enhanced respiratory recovery on the operative side.

Various analgesic modalities are available for postoperative pain control following thoracotomy, including oral analgesics, epidural anesthesia, intercostal nerve blocks, and opioid administration.^8)^ Each of these methods has inherent advantages and limitations. For example, epidural anesthesia carries the risk of epidural hematoma, particularly in the context of systemic heparinization. Similarly, intercostal nerve blocks have a limited duration of effect, and opioid usage can lead to respiratory depression, delirium, constipation, and reduced activity levels, which might delay postoperative rehabilitation.

Intercostal nerve cryoablation causes reversible axonotmesis of the intercostal nerves, allowing for prolonged analgesia over a period of weeks to months.^11)^ Although potential complications include chest wall hypoesthesia, persistent neuropathic pain, and bleeding from intercostal arteries, several reports indicate that sensation to the chest wall typically returns over time and that long-term neuropathic pain is rare.^4)^ Moreover, bleeding from intercostal arteries can be minimized through careful inspection and hemostasis of the chest wall at the end of surgery. Therefore, in aortic surgery via left thoracotomy—where effective pain control is essential and dissection is already extensive—cryoablation might offer a favorable risk–benefit profile.

CT-based lung volume measurement is a reliable method for objectively assessing the effectiveness of analgesia. Effective postoperative pain control has long been recognized as a key factor in reducing pulmonary complications following thoracic surgery.^5,6)^ In our study, the cryoablation group demonstrated a significantly higher left lung volume ratio at 1 week postoperatively compared to the non-cryoablation group, indicating better pulmonary expansion on the operative side. Although pain can inhibit respiratory motion bilaterally and does not inherently result in asymmetrical lung volume reduction, we believe that the observed lateralized effect may be explained by improved pain control with cryoablation. More specifically, effective analgesia likely facilitated stronger coughing and better engagement in physiotherapy, which in turn may have helped prevent postoperative atelectasis in the left lung. As a result, lung volume was better preserved on the affected side.

Despite its promising findings, this study has several limitations. First, it was a retrospective observational study conducted at a single center with a relatively small sample size (n = 62). However, we performed a post hoc power analysis for the primary outcome. With a mean difference in pain scores of 0.7 and a pooled standard deviation of 0.9, the estimated statistical power was 0.86 (α = 0.05, 2-tailed), indicating that the study was sufficiently powered to detect a clinically meaningful difference. In addition, statistically significant differences were observed in key clinical outcomes, including pain scores, opioid consumption, and postoperative lung volume ratios. These results support the clinical utility of intercostal nerve cryoablation in thoracic aortic surgery. Furthermore, the use of objective CT-based lung volume assessment strengthens the reliability of the evaluation of postoperative pain management strategies. Prospective studies with larger cohorts are warranted to validate our findings and to further refine perioperative analgesic protocols in this setting.

Second, while CT-based lung volume measurements were used as a surrogate for pulmonary function, spirometry would have offered a more direct and standardized evaluation. Unfortunately, postoperative spirometry was not performed uniformly across all patients, which limited its inclusion in the current analysis. Similarly, the duration of oxygen therapy could not be consistently assessed across the cohort due to variability in clinical documentation and transfers to other hospitals. Therefore, this parameter was also excluded from the present analysis.

Third, there was a significant difference in the proportion of smokers between the 2 groups, which may have influenced postoperative pulmonary outcomes. This disparity has been acknowledged as a limitation of the study.

Finally, phrenic nerve palsy is another important factor influencing postoperative lung volume. However, this parameter was not assessed in the present study because, in most cases, the diaphragm was partially or completely incised and subsequently repaired. Such repair often results in the diaphragm being fixed in a lower position on the surgical side due to shortening at the suture line, making classic radiographic signs of phrenic nerve palsy—such as diaphragmatic elevation—difficult to detect on postoperative imaging. Therefore, a new method for evaluating diaphragmatic function may be warranted in future studies.

## Conclusion

Intercostal nerve cryoablation is a safe and effective technique for managing postoperative pain following left thoracotomy for aortic repair. Since it reduces opioid consumption and facilitates postoperative physiotherapy, its incorporation into surgical protocols may improve clinical outcomes, particularly in patients at risk for respiratory complications.

## Declarations

### Data availability

The datasets generated and/or analyzed during the current study are available from the corresponding author on reasonable request.

## References

[1] Minatoya K, Sato Y, Toh Y, et al. Thoracic and cardiovascular surgeries in Japan during 2019: Annual report by the Japanese Association for Thoracic Surgery. Gen Thorac Cardiovasc Surg 2023; 71: 595–628. 37470949 10.1007/s11748-023-01945-4 PMC10509063

[2] Wenk M, Schug SA. Perioperative pain management after thoracotomy. Curr Opin Anaesthesiol 2011; 24: 8–12. 21084984 10.1097/ACO.0b013e3283414175

[3] Marshall K, McLaughlin K. Pain management in thoracic surgery. Thorac Surg Clin 2020; 30: 339–46. 32593366 10.1016/j.thorsurg.2020.03.001

[4] Lai K, Eldredge RS, Zobel M, et al. Intercostal nerve cryoablation for postoperative pain control in pediatric thoracic surgery: a scoping review. J Laparoendosc Adv Surg Tech A 2023; 33: 994–1004. 37462727 10.1089/lap.2023.0070

[5] Kehlet H, Dahl JB. The value of “multimodal” or “balanced analgesia” in postoperative pain treatment. Anesth Analg 1993; 77: 1048–56. 8105724 10.1213/00000539-199311000-00030

[6] Chou R, Gordon DB, de Leon-Casasola OA, et al. Management of Postoperative Pain: A Clinical Practice Guideline from the American Pain Society, the American Society of Regional Anesthesia and Pain Medicine, and the American Society of Anesthesiologists’. Committee on Regional Anesthesia, Executive Committee, and Administrative Council. J Pain 2016; 17: 131–57. 10.1016/j.jpain.2015.12.008 26827847

[7] Soto RG, Fu ES. Acute pain management for patients undergoing thoracotomy. Ann Thorac Surg 2003; 75: 1349–57. 12683601 10.1016/s0003-4975(02)04647-7

[8] Daemen JHT, de Loos ER, Vissers YLJ, et al. Intercostal nerve cryoablation versus thoracic epidural for postoperative analgesia following pectus excavatum repair: a systematic review and meta-analysis. Interact Cardiovasc Thorac Surg 2020; 31: 486–98. 32929487 10.1093/icvts/ivaa151

[9] Eldredge RS, McMahon L. Intercostal nerve cryoablation therapy for the repair of pectus excavatum: a systematic review. Front Surg 2023; 10: 1235120. 37693640 10.3389/fsurg.2023.1235120 PMC10484532

[10] Maxwell CM, Weksler B, Houda J, et al. Intercostal cryoablation during video-assisted lung resection can decrease postoperative opioid use. Innovations (Phila) 2023; 18: 352–6. 37461202 10.1177/15569845231185583

[11] Gage AA, Baust J. Mechanisms of tissue injury in cryosurgery. Cryobiology 1998; 37: 171–86. 9787063 10.1006/cryo.1998.2115

